# COVID-19 and Spillover Effect of Global Economic Crisis on the United States’ Financial Stability

**DOI:** 10.3389/fpsyg.2021.632175

**Published:** 2021-02-26

**Authors:** Khurram Shehzad, Liu Xiaoxing, Faik Bilgili, Emrah Koçak

**Affiliations:** ^1^School of Economics and Management, Southeast University, Nanjing, China; ^2^Department of Economics, Faculty of Economics and Administrative Sciences, Erciyes University, Melikgazi, Turkey

**Keywords:** COVID-19 and financial stability, worldwide economic crisis, fear gage, economic sustainability, ARDL model

## Abstract

Due to the novel coronavirus pandemic (COVID-19), the lockdown engendered has had a vicious impact on the global economy. This analysis’ prime intention is to evaluate the impact of the United States’ economic and health crisis as a result of COVID-19 on its financial stability. Additionally, the investigation analyzed the spillover impact of the worldwide economic slowdown experienced by COVID-19 on the United States’ financial volatility. The study applied an autoregressive distributed lag (ARDL) model and discovered that the economic and health crises that occurred in the United States portentously upset the future expectations of its investors. Conspicuously, the health crisis in Spain and Italy were ominous spillovers of the United States’ financial instability in the short-run. Likewise, an economic crisis ensued in the United Kingdom because of COVID-19 causing spillover for the United States markets’ financial instability. The examination evaluated that Asian and African nations’ economic crises perilously affects the United States’ financial stability. The study determined that financial instability occurred in the United States due to its own economic and health crises persisted for a longer period than financial disequilibrium that occurred in other nations. The analysis suggested some strategies of smart lockdown that the government of the United States and other nations should follow to restart the economic cycle through tighter controls to minimize losses by following the steps of (a) preparing a lockdown checklist, (b) monitoring completion of lockdown tasks, and (c) complete a close-down stock take or count.

## Introduction

To date, the World Health Organization (WHO) has taken various initiatives against several viral infections (WHO, 2020). However, a few emerged to such escalated levels that could ravage economic infrastructure due to deadly lockdowns (Gladstone, 2020). The most well-known among the proclaimed pandemics include the influenza outbreak that appeared in 1918, severe acute respiratory syndrome (SARS) in 2002, and Ebola in 2013. These were deadly and devastating to social and economic infrastructure all around the globe (Gaffen, 2020; Fernandes, 2020). For instance, the outbreak of influenza marked an overwhelming infection rate of one-third of the world population (Crosby, 2003). Second, SARS emerged in Guangdong, China and rapidly spread across Asian countries and their surroundings, it infected more than 8000 people, and consequently, the SARS epidemic caused the death of more than 900 people (Peiris et al., 2003).

Finally, Ebola became the third viral pandemic disease to generate a death toll of more than 11,300 people and caused a loss of $53 billion for the United States (Fernandes, 2020). Currently, the world is encountering a new viral pandemic known as novel coronavirus 2019 (COVID-19), which started in Wuhan, China, in December 2019, and reached other parts of the world very rapidly (Albulescu, 2020; Ashraf, 2020; Béland et al., 2020). In mid-March 2020, Europe became the new epicenter of COVID-19, beginning in Italy, the infection spread into the neighboring countries and then out to almost all parts of the world. It had infected 2,471,930 people, and 170,129 deaths had been reported worldwide by April 21, 2020 (Financial Times, 2020). The ease of transmission of COVID-19 from one person to another formed an emergency worldwide (Huang et al., 2020; Salisu and Akanni, 2020). As a result, numerous firms were compelled to temporarily halt their business activities.

**FIGURE 1:**
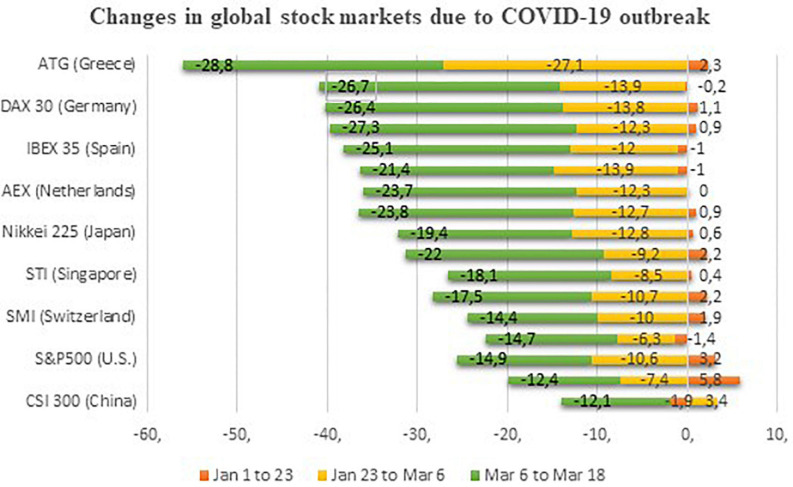
Decline in financial markets due to COVID-19. Source: Statista (2020).

**FIGURE 2:**
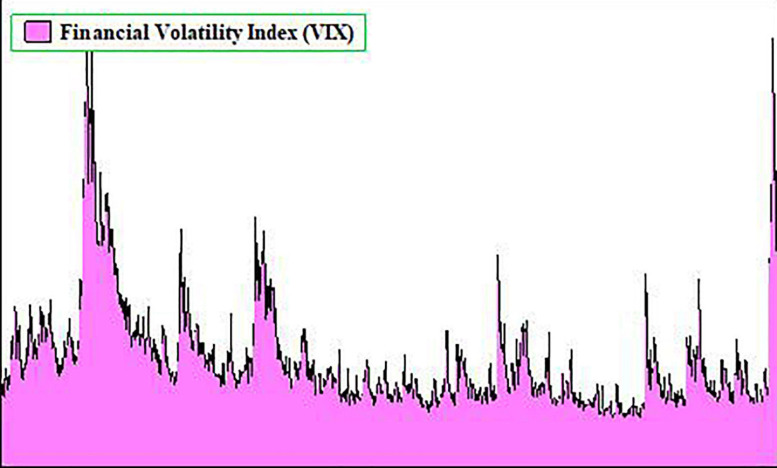
Financial volatility index since 2007. Source: Chicago Board Options Exchange [CBOE] (2020).

Indeed, unemployment and inflation increased, while economic growth, tourism, and sales through online travel agencies (OTAs), hotels, and airlines fell suddenly throughout the globe (ILO, 2020; Leduc and Liu, 2020; Gössling et al., 2021). The economic shocks felt during the COVID-19 outbreak are estimated to be more dangerous than the economic shocks in SARS (Ayittey et al., 2020; World Economic Forum [WEF], 2020). A market diagnosis accomplished by Bloomberg concluded that COVID-19 has reduced China’s first quarter of GDP growth by about 4.5% (Bocca, 2020). The effects of COVID-19 has also reshaped financial markets worldwide (Daube, 2020; Goodell, 2020; Mazur et al., 2020), and the market value of the United States Standard & Poor Index (S&P 500) has plunged to almost 30% since the outbreak of COVID-19 in the United States (USC) (Yahoo, 2020). Figure 1 shows that the S&P 500 and Nasdaq index’s market value had fallen by 14.9 and 12.4% from March 6 – March 18, respectively. However, other reputed markets of the world, i.e., DAX 30, Nikkei 225, FTSE 100, CAC 40, and CSI 300 had also suffered a deterioration of 25.4, 19.4, 21.4, 26.7, and 12.1% in their values due to the outbreak of COVID-19, respectively. Recently, the United States’ 10-year treasury yield index encountered the highest decline of its history (Tarigan, 2020). Likewise, Figure 2 below shows that the day-wise series of the financial volatility index (VIX) has upturned numerous times since 2008. The financial VIX, also known as “fear gauge,” generated by Chicago Board Options Exchange (CBOE), is a reliable source to determine the level of financial stability (Chicago Board Options Exchange [CBOE], 2020). The index offers 30-day predictions for investors related to the volatility of the S&P 500 index (Leduc and Liu, 2020). It shot up to its highest level in the era of global financial crises (GFC) in 2007–2009. Similarly, the VIX also jumped at the time of the trade war between the United States and China (2018). However, in recent times, the outbreak of COVID-19 and its impact on the economy and stock markets increased the level of the VIX. Its highest point was equal to that of the GFC (2007–2009) (Just and Krzysztof, 2020; Sharif et al., 2020). Consequently, this investigation employed the VIX as a critical tool to measure the United States’ financial volatility. Besides, this information shows that the financial stability of the United States has been severely affected by COVID-19.

This project set out to investigate the impact of COVID-19 on the financial stability of the United States. This study mainly evaluates the economic crisis and health crisis that took place because of USC. Furthermore, the examination recognized the spillover effect of economic and health crises, due to COVID-19 in the United States, Europe, Asia, and Africa, on the United States’ financial volatility. The research utilized the data of confirmed cases as the nomination of lockdown condition and number of deaths because of COVID-19 as the nomination of the health crisis. The study stated that as the number of confirmed cases of COVID-19 increased, the lockdown condition became stricter, which led to a significant economic crisis. Additionally, deaths that happened due to COVID-19 indicate the lack of medical facilities and control in the pandemic, which led to a significant health crisis. The contribution of the paper to the literature can be expressed as follows:

This examination answered the imperious queries of researchers, academicians, policymakers, and investors. For instance, first, does the lockdown and health crisis in the United States expressively distress its financial stability? Second, do economic crises have a higher impact on financial stability than health crises? Third, does economic crisis in European nations and in the United States have the same effect on United States financial instability? Fourth, do economic and health crises in Asian countries spillover to the United States’ financial vulnerability? Fifth, how does economic uncertainty move with COVID-19 fear in Arab nations? Sixth, how can the financial stability of the United States be saved from harmful waves of COVID-19? The author believes that answers to these questions will make clear the role of economic crisis and health emergencies, due to COVID-19 in the United States, European, Arabian, African, and Asian countries, for the United States’ financial depression. Furthermore, a nation-wise strategic plan can be generated for their economic and financial sustainability.

Initially, this study ensured the appropriateness of the variables used with the help of two methodologies, the Phillip Perron (PP) test and the Augmented Dickey-Fuller (ADF) test. The investigation described the variables that had a varying level of integration, i.e., I(0), I(1), and no variable found to be stationary at I(2). Accordingly, an autoregressive distributed lag (ARDL) model was employed to ascertain the long- and short-run affiliation among variables.

The rest of this study is structured as follows: section “Data and Methodology” provides details on the analytical approach used in this investigation. Section “Findings and Interpretations” delivers a debate on results and a discussion of this investigation. Section “Conclusion” offers the conclusion, research implications, and limitations.

## Data and Methodology

### Data

This investigation has utilized the daily data of the confirmed number of patients infected, the number of deaths due to COVID-19, and the VIX from December 31, 2019 to April 10, 2020. The data used in this investigation are retrieved from the European Centre for Disease Prevention and Control, and the Yahoo Finance database.

### Model Specification

This study analyzed the impact of COVID-19 in the United States, Europe, Asia, Africa, and Arabian nations, and divided the dataset into sub-sections^1^, i.e., Italy, Spain, Germany, and the United Kingdom as model A; the United States, Switzerland, the Netherlands, and France as a model B, China, Iran, Japan, and Pakistan as model C, Saudi Arabia, United Arab Emirates (UAE), and Qatar as model D, and Egypt, Morocco, Kenya, Tunisia, and Ghana as model E. All these sections cannot be regressed in one equation due to the degree of freedom’s complexity. Hence, these nations were divided into different sections. Moreover, these sections will also help generate a more enhanced nation-wise conclusion about the impact of COVID-19 on the United States’ financial stability. The functional form of these sections can be written as follows:

(1) V⁢I⁢X=f⁢(I⁢T⁢C,I⁢T⁢D,S⁢P⁢C,S⁢P⁢D,G⁢R⁢C,G⁢R⁢D,U⁢K⁢C,U⁢K⁢D)

(2) V⁢I⁢X=f⁢(U⁢S⁢C,U⁢S⁢D,S⁢W⁢C,S⁢W⁢D,N⁢E⁢T⁢C,N⁢E⁢T⁢D,F⁢R⁢C,F⁢R⁢D)

(3) V⁢I⁢X=f⁢(C⁢H⁢C,C⁢H⁢D,I⁢R⁢C,I⁢R⁢D,J⁢A⁢P⁢C,J⁢A⁢P⁢D,P⁢A⁢K⁢C)

(4) V⁢I⁢X=f⁢(S⁢A⁢C,U⁢A⁢E⁢C,Q⁢T⁢R⁢C)

(5) V⁢I⁢X=f⁢(E⁢G⁢P⁢C,M⁢O⁢R⁢C,K⁢E⁢N⁢C,T⁢U⁢N⁢C,G⁢H⁢N⁢C)

Here in Eq. 1, ITC, ITD, SPC, SPD, GRC, GRD, UKC, and UKD represent the confirmed number of patients of COVID-19 in Italy, the confirmed number of deaths due to COVID-19 in Italy, confirmed number of patients in Spain, confirmed number of deaths in Spain, confirmed number of patients in Germany, confirmed number of deaths in Germany, confirmed number of patients in the United Kingdom, and confirmed number of deaths in the United Kingdom, respectively. In Eq. 2, USC denotes the confirmed number of cases in the United States, USD represents the confirmed deaths in the United States, SWC represents confirmed cases in Switzerland, SWD represents the deaths in Switzerland, NETC represents confirmed patients in the Netherlands, NETD represents deaths in the Netherlands, FRC denotes the confirmed cases in France, and FRD denotes the number of deaths in France due to COVID-19. Moreover, Eq. 3 defines model C; here, CHC, CHD, IRC, IRD, JAPC, JAPD, and PAKC denote the confirmed number of patients in China, deaths in China, confirmed patients in Iran, deaths in Iran, confirmed patients in Japan, deaths in Japan, and confirmed cases in Pakistan, respectively; while Eq. 4 denotes the Arabian section. Here, SAC, UAEC, and QTRC denote the confirmed patients of COVID-19 in Saudi Arabia, UAE, and Qatar. At last, Eq. 5 represents section E (African nations); where, EGPC, MORC, KENC, TUNC, and GHNC represent the confirmed number of patients of COVID-19 in Egypt, Morocco, Kenya, Tunisia, and Ghana.

### An Autoregressive Distributed Lag Estimation

Our study used an ARDL model introduced by Pesaran et al. (2001) to ascertain the long- and short-run affiliation among variables used in this examination. The ARDL model is suitable for a small number of observations. Moreover, it can be employed to assess the exact parameters; either the variables are stationary at level I(0), at the first difference I(1), or an amalgamation of both. Furthermore, long-run and short-run coefficients can be determined at the same time. The cointegration equations for the above-defined sections can be described through the ARDL bound testing approach as follows:

(6) $$\begin{array}{c} \text{Δ}VI\;X_{t}={\text{ζ}}_{0}+{\text{ζ}}_{1}\overset{p}{\underset{i=1}{\sum }}\text{Δ}VIX_{t-i}+{\text{ζ}}_{2}\overset{p}{\underset{i=1}{\sum }}\text{Δ}ITC_{t-i}+{\text{ζ}}_{3}\overset{p}{\underset{i=1}{\sum }}\text{Δ}ITD_{t-i}\\ +{\text{ζ}}_{4}\overset{p}{\underset{i=1}{\sum }}\text{Δ}SPC_{t-i}+{\text{ζ}}_{5}\overset{p}{\underset{i=1}{\sum }}\text{Δ}SPD_{t-i}+{\text{ζ}}_{6}\overset{p}{\underset{i=1}{\sum }}\text{Δ}GRC_{t-i}\\ +{\text{ζ}}_{7}\overset{p}{\underset{i=1}{\sum }}\text{Δ}GRD_{t-i}+{\text{ζ}}_{8}\overset{p}{\underset{i=1}{\sum }}\text{Δ}UKC_{t-i}+{\text{ζ}}_{9}\overset{p}{\underset{i=1}{\sum }}\text{Δ}UKD_{t-i}\\ +{\text{φ}}_{1}VIX_{t-1}+{\text{φ}}_{2}ITC_{t-1}+{\text{φ}}_{3}ITD_{t-1}+{\text{φ}}_{4}SPC_{t-1}\\ +{\text{φ}}_{5}SPD_{t-1}+{\text{φ}}_{6}GRC_{t-1}+{\text{φ}}_{7}GRD_{t-1}\\ +{\text{φ}}_{8}UKC_{t-1}+{\text{φ}}_{9}UKD_{t-1}+{\text{μ}}_{t} \end{array}$$

(7) $$\begin{array}{c} \text{Δ}VIX_{t}={\text{ζ}}_{0}+{\text{ζ}}_{1}\overset{p}{\underset{i=1}{\sum }}\text{Δ}VIX_{t-i}+{\text{ζ}}_{2}\overset{p}{\underset{i=1}{\sum }}\text{Δ}USC_{t-i}+{\text{ζ}}_{3}\overset{p}{\underset{i=1}{\sum }}\text{Δ}USD_{t-i}\\ +{\text{ζ}}_{4}\overset{p}{\underset{i=1}{\sum }}\text{Δ}SWC_{t-i}+{\text{ζ}}_{5}\overset{p}{\underset{i=1}{\sum }}\text{Δ}SWD_{t-i}+{\text{ζ}}_{6}\overset{p}{\underset{i=1}{\sum }}\text{Δ}NETC_{t-i}\\ +{\text{ζ}}_{7}\overset{p}{\underset{i=1}{\sum }}\text{Δ}NETD_{t-i}+{\text{ζ}}_{8}\overset{p}{\underset{i=1}{\sum }}\text{Δ}FRC_{t-i}+{\text{ζ}}_{9}\overset{p}{\underset{i=1}{\sum }}\text{Δ}FRD_{t-i}\\ +{\text{φ}}_{1}VIX_{t-1}+{\text{φ}}_{2}USC_{t-1}+{\text{φ}}_{3}USD_{t-1}+{\text{φ}}_{4}SWC_{t-1}\\ +{\text{φ}}_{5}SWD_{t-1}+{\text{φ}}_{6}NETC_{t-1}+{\text{φ}}_{7}NETD_{t-1}\\ +{\text{φ}}_{8}FRC_{t-1}+{\text{φ}}_{9}FRD_{t-1}+{\text{μ}}_{t} \end{array}$$

(8) $$\begin{array}{c} \text{Δ}VIX_{t}={\text{ζ}}_{0}+{\text{ζ}}_{1}\overset{p}{\underset{i=1}{\sum }}\text{Δ}VIX_{t-i}+{\text{ζ}}_{2}\overset{p}{\underset{i=1}{\sum }}\text{Δ}CHC_{t-i}+{\text{ζ}}_{3}\overset{p}{\underset{i=1}{\sum }}\text{Δ}CHD_{t-i}\\ +{\text{ζ}}_{4}\overset{p}{\underset{i=1}{\sum }}\text{Δ}IRC_{t-i}+{\text{ζ}}_{5}\overset{p}{\underset{i=1}{\sum }}\text{Δ}IRD_{t-i}+{\text{ζ}}_{6}\overset{p}{\underset{i=1}{\sum }}\text{Δ}JAPC_{t-i}\\ +{\text{ζ}}_{7}\overset{p}{\underset{i=1}{\sum }}\text{Δ}JAPD_{t-i}+{\text{ζ}}_{8}\overset{p}{\underset{i=1}{\sum }}\text{Δ}PAKC_{t-i}+{\text{φ}}_{1}VIX_{t-1}\\ +{\text{φ}}_{2}CHC_{t-1}+{\text{φ}}_{3}CHD_{t-1}+{\text{φ}}_{4}IRC_{t-1}+{\text{φ}}_{5}IRD_{t-1}\\ +{\text{φ}}_{6}JAPCC_{t-1}+{\text{φ}}_{7}JAPD_{t-1}+{\text{φ}}_{8}PAKC_{t-1}+{\text{μ}}_{t} \end{array}$$

(9) $$\begin{array}{c} \text{Δ}VIX_{t}={\text{ζ}}_{0}+{\text{ζ}}_{1}\overset{p}{\underset{i=1}{\sum }}\text{Δ}VIX_{t-i}+{\text{ζ}}_{2}\overset{p}{\underset{i=1}{\sum }}SAC_{t-i}\\ +{\text{ζ}}_{3}\overset{p}{\underset{i=1}{\sum }}\text{Δ}UAEC_{t-i}+{\text{ζ}}_{4}\overset{p}{\underset{i=1}{\sum }}\text{Δ}QTRC_{t-i}\\ +{\text{φ}}_{1}VIX_{t-1}+{\text{φ}}_{2}SAC_{t-1}+{\text{φ}}_{3}UAEC_{t-1}+{\text{φ}}_{4}QTRC_{t-1}+{\text{μ}}_{t} \end{array}$$

(10) $$\begin{array}{c} \text{Δ}VIX_{t}={\text{ζ}}_{0}+{\text{ζ}}_{1}\overset{p}{\underset{i=1}{\sum }}\text{Δ}VIX_{t-i}+{\text{ζ}}_{2}\overset{p}{\underset{i=1}{\sum }}\text{Δ}EGPC_{t-i}\\ +{\text{ζ}}_{3}\overset{p}{\underset{i=1}{\sum }}\text{Δ}MORC_{t-i}+{\text{ζ}}_{4}\overset{p}{\underset{i=1}{\sum }}\text{Δ}KENC_{t-i}\\ +{\text{ζ}}_{5}\overset{p}{\underset{i=1}{\sum }}\text{Δ}TUNC_{t-i}+{\text{ζ}}_{6}\overset{p}{\underset{i=1}{\sum }}\text{Δ}GHNC_{t-i}+{\text{φ}}_{1}VIX_{t-1}\\ +{\text{φ}}_{2}EGPC_{t-1}+{\text{φ}}_{3}MORC_{t-1}+{\text{φ}}_{4}KENC_{t-1}\\ +{\text{φ}}_{5}TUNC_{t-1}+{\text{φ}}_{6}GHNC_{t-1}+{\text{μ}}_{t} \end{array}$$

Where △ denotes the first difference, and *i* represents the number of lag values. Further, ζ and φ signify the short- and long-run coefficients, respectively; while, ζ_0_ indicates the intercept parameter and μ_*t*_means residual factor. The ARDL model relies on the F-test technique to conclude whether the long-run cointegration between variables exists or not. Pesaran et al. (2001) specified the two bounds, i.e., lower and upper bound. Suppose the computed statistics of the F-test are greater than the upper bound. In that case, the null hypothesis of no cointegration exists is efficaciously rejected, which identifies the long-run affiliation between study variables. However, if the F-test value is less than the lower bound, then the null hypothesis of the ARDL model cannot be rejected. Besides, if the F-test value remains between the upper and lower bound limits, then the model is inconclusive. In order to evaluate the short-run ties between the study variables, an ARDL based error correction model (ECM) can be specified as follows:

(11) $$\begin{array}{c} \text{Δ}VIX_{t}={\text{ζ}}_{0}+{\text{ζ}}_{1}\overset{p}{\underset{i=1}{\sum }}\text{Δ}VIX_{t-i}+{\text{ζ}}_{2}\overset{p}{\underset{i=1}{\sum }}\text{Δ}ITC_{t-i}\\ +{\text{ζ}}_{3}\overset{p}{\underset{i=1}{\sum }}\text{Δ}ITD_{t-i}+{\text{ζ}}_{4}\overset{p}{\underset{i=1}{\sum }}\text{Δ}SPC_{t-i}+{\text{ζ}}_{5}\overset{p}{\underset{i=1}{\sum }}\text{Δ}SPD_{t-i}\\ +{\text{ζ}}_{6}\overset{p}{\underset{i=1}{\sum }}\text{Δ}GRC_{t-i}+{\text{ζ}}_{7}\overset{p}{\underset{i=1}{\sum }}\text{Δ}GRD_{t-i}+{\text{ζ}}_{8}\overset{p}{\underset{i=1}{\sum }}\text{Δ}UKC_{t-i}\\ +{\text{ζ}}_{9}\overset{p}{\underset{i=1}{\sum }}\text{Δ}UKD_{t-i}+{\text{ψ}}_{1}ECT_{t-1}+{\text{μ}}_{t} \end{array}$$

(12) $$\begin{array}{c} \text{Δ}VIX_{t}={\text{ζ}}_{0}+{\text{ζ}}_{1}\overset{p}{\underset{i=1}{\sum }}\text{Δ}VIX_{t-i}+{\text{ζ}}_{2}\overset{p}{\underset{i=1}{\sum }}\text{Δ}USC_{t-i}\\ +{\text{ζ}}_{3}\overset{p}{\underset{i=1}{\sum }}\text{Δ}USD_{t-i}+{\text{ζ}}_{4}\overset{p}{\underset{i=1}{\sum }}\text{Δ}SWC_{t-i}+{\text{ζ}}_{5}\overset{p}{\underset{i=1}{\sum }}\text{Δ}SWD_{t-i}\\ +{\text{ζ}}_{6}\overset{p}{\underset{i=1}{\sum }}\text{Δ}NETC_{t-i}+{\text{ζ}}_{7}\overset{p}{\underset{i=1}{\sum }}\text{Δ}NETD_{t-i}+{\text{ζ}}_{8}\overset{p}{\underset{i=1}{\sum }}\text{Δ}FRC_{t-i}\\ +{\text{ζ}}_{9}\overset{p}{\underset{i=1}{\sum }}\text{Δ}FRD_{t-i}+{\text{ψ}}_{1}ECT_{t-1}+{\text{μ}}_{t} \end{array}$$

(13) $$\begin{array}{c} \text{Δ}VIX_{t}={\text{ζ}}_{0}+{\text{ζ}}_{1}\overset{p}{\underset{i=1}{\sum }}\text{Δ}VIX_{t-i}+{\text{ζ}}_{2}\overset{p}{\underset{i=1}{\sum }}\text{Δ}CHC_{t-i}\\ +{\text{ζ}}_{3}\overset{p}{\underset{i=1}{\sum }}\text{Δ}CHD_{t-i}+{\text{ζ}}_{4}\overset{p}{\underset{i=1}{\sum }}\text{Δ}IRC_{t-i}\\ +{\text{ζ}}_{5}\overset{p}{\underset{i=1}{\sum }}\text{Δ}IRD_{t-i}+{\text{ζ}}_{6}\overset{p}{\underset{i=1}{\sum }}\text{Δ}JAPC_{t-i}\\ +{\text{ζ}}_{7}\overset{p}{\underset{i=1}{\sum }}\text{Δ}JAPD_{t-i}+{\text{ζ}}_{8}\overset{p}{\underset{i=1}{\sum }}\text{Δ}PAKC_{t-i}+{\text{ψ}}_{1}ECT_{t-1}+{\text{μ}}_{t} \end{array}$$

(14) $$\begin{array}{c} \text{Δ}VIX_{t}={\text{ζ}}_{0}+{\text{ζ}}_{1}\overset{p}{\underset{i=1}{\sum }}\text{Δ}VIX_{t-i}+{\text{ζ}}_{2}\overset{p}{\underset{i=1}{\sum }}\text{Δ}SAC_{t-i}\\ +{\text{ζ}}_{3}\overset{p}{\underset{i=1}{\sum }}\text{Δ}UAEC_{t-i}+{\text{ζ}}_{4}\overset{p}{\underset{i=1}{\sum }}\text{Δ}QTRC_{t-i}+{\text{ψ}}_{1}ECT_{t-1}+{\text{μ}}_{t} \end{array}$$

(15) $$\begin{array}{c} \text{Δ}VIX_{t}={\text{ζ}}_{0}+{\text{ζ}}_{1}\overset{p}{\underset{i=1}{\sum }}\text{Δ}VIX_{t-i}+{\text{ζ}}_{2}\overset{p}{\underset{i=1}{\sum }}\text{Δ}EGPC_{t-i}\\ +{\text{ζ}}_{3}\overset{p}{\underset{i=1}{\sum }}\text{Δ}MORC_{t-i}+{\text{ζ}}_{4}\overset{p}{\underset{i=1}{\sum }}\text{Δ}KENC_{t-i}+{\text{ζ}}_{5}\overset{p}{\underset{i=1}{\sum }}\text{Δ}TUNC_{t-i}\\ +{\text{ζ}}_{6}\overset{p}{\underset{i=1}{\sum }}\text{Δ}GHNC_{t-i}+{\text{ψ}}_{1}ECT_{t-1}+{\text{μ}}_{t} \end{array}$$

Here, the error correction term (ECT_*t–1*_) indicates the adjustment speed of VIX to accomplish long-run equilibrium. This examination evaluates the stability of the ARDL model with the use of serial correlation and a heteroskedasticity test. Simultaneously, the cumulative sum of recursive residuals (CUSUM) and the square of the cumulative sum of the recursive residuals (CUSUMSQ) test is also applied.

## Findings and Interpretations

### Summary Statistics, Unit Root, and Long-Run Cointegration Analysis

The summary statistics given in Table 1 shows that the average number of confirmed patients was high in the United States and Italy. In contrast, average deaths due to COVID-19 were high in Italy and Spain. It indicates that the United States and Italy are the two biggest nations affected by COVID-19. The Jarque–Bera values denote that the study variables were not normally distributed; however, this problem can be removed through the ARDL model. Table 2 exhibits the results of the ADF test (Dickey and Fuller, 1979) and the PP test (Phillips and Perron, 1988). The outcomes declared that the study variables had a mixed stationary level, i.e., I(0) and I(1), but no one variable was stationary at I(2). Hence, the ARDL technique can be exercised to ascertain the cointegration and long- and short-run parameters. The ARDL model has critical capabilities compared to other regression models; first, it is suitable for a small data set. Second, it can be used for a different level of cointegrated factors. Third, it explained the long-and short-run coefficients after considering its previous values and past figures of independent factors as well. After exploring the unit root level of the study variables, the next step was to determine the long-run cointegration between the study variables using the ARDL model.

**TABLE 1:** Descriptive statistics of study variables.

	VIX	UKD	UKC	SPD	SPC	ITD	ITC	GRD
Mean	35.26	14.00	238.79	88.04	1061	142.4	13876	5.95
Skewness	0.614	3.679	2.697	2.45	2.23	1.50	1.218	2.65
Kurtosis	1.884	17.86	9.659	7.88	6.90	3.78	2.836	9.03
Jarque–Bera	5.626	561.5	149.9	98.03	71.8	19.78	12.17	131
Probability	0.06	0.000	0.000	0.00	0.00	0.00	0.002	0.0
	GRC	USD	USC	SWD	SWC	NETD	NETC	FRD
Mean	770.8	28.59	1843	3.122	182.7	10.16	140.9	36.0
Skewness	2.182	2.723	2.56	4.457	1.68	3.085	2.18	2.5
Kurtosis	6.351	9.199	8.428	24.78	4.15	12.51	6.71	8.5
Jarque–Bera	61.84	139.0	113.7	1131.	26.060	262.4	66.9	115
Probability	0.000	0.000	0.000	0.000	0.000	0.000	0.00	0.00
	FRC	CHC	CHD	IRC	IRD	JAPC	JAPD	PAKC
Mean	536.7	1308	51.30	619.2	46.12	27.8	1.00	28.4
Skewness	2.07	3.97	1.715	1.24	0.79	2.13	1.322	1.5
Kurtosis	6.37	21.30	6.60	4.05	1.90	8.88	3.66	3.7
Jarque–Bera	58.48	663.7	41.22	12.1	6.271	88.08	12.39	17.2
Probability	0.000	0.000	0.00	0.00	0.04	0.000	0.00	0.00
	QTRC	SAC	UAEC	EGPC	GHNC	KENC	MORC	TUNC
Mean	27.20	43.0	15.45	23.9	8.93	2.46	23.53	13.2
Skewness	3.431	1.540	1.98	1.20	2.58	1.13	2.085	1.68
Kurtosis	14.23	4.497	6.465	4.25	8.83	2.96	6.803	4.47
Jarque–Bera	144.4	9.782	23.1	4.585	38.0	3.19	19.91	8.45
Probability	0.000	0.007	0.000	0.100	0.00	0.20	0.000	0.01

*Author’s calculation.*

**TABLE 2:** Findings of unit root methodology.

	Variables	VIX	EGPC	KENC	TUNC	GHNC	MORC	SAC	UAEC	QTRC	CHC
ADF	Level	−2.45	−2.75	−1.37	−0.92	3.21*	4.8*	−2.12	−3.4*	−3.7*	−2.71
	1st difference	−6***	−5.6***	−3.03*	−4.0***	−9.9***	−2.5	−6***	−3.6*	−6***	6.8***
PP	Level	−3.15	−2.71	−4.69*	−2.891	−2.75	12.0***	−2.10	−3.0*	−3.7*	−3.9*
	1st difference	−5.8**	−9.85***	−15***	−15.3***	−8.2***	−3.74*	−6***	−6.1***	−9***	22***
	Variables	CHD	IRC	IRD	JAPC	JAPD	PAKC	GRC	GRD	ITC	ITD
ADF	Level	−2.64	3.26*	−0.169	0.92	−3.29*	−0.29	1.0	22.9***	1.3	−3.8*
	1st difference	−1.01	−3.99*	−4.61*	−4.156*	−8.5***	−7.4***	−16***	−3.4*	−9***	−5***
PP	Level	−3.48*	3.07*	−0.23	−4.16*	−3.208*	−2.4	−1.34	3.2*	0.05*	2.6
	1st difference	11***	−4.21**	−4.61**	−3.80*	−23***	−11***	−11***	−7.5***	10***	−9***
	Variables	SPC	SPD	UKC	UKD	USC	USD	SWC	SWD	FRC	FRD
ADF	Level	4.42*	12.26***	7.3***	−24***	7.6***	7.2***	4.5*	6.5***	−0.30	4.2**
	1st difference	−3.4*	−3.46*	−1.55	−6.64***	−5***	−2.7***	−13***	−14***	−10***	−3.9*
PP	Level	0.87	4.868**	12.6***	12.7***	5.6***	4.1***	−0.8	−3.9*	−2.24	0.1
	1st difference	−2.87	−8.58***	−4.72*	−4.37*	−5***	−8.4***	−12***	−13***	−15***	−9***

*, **, and *** denotes the significance level at 10, 5, and 1%, respectively.Author’s calculation.

This study employed lags based on Akaike Information Criterion to adopt the best regression model. Table 3 displays the consequences of the ARDL bound test for each equation. The F-statistics values, 19.3, 8.7, 6.1, 8.61, and 8.67 for Eq. 6, Eq. 7, Eq. 8, Eq. 9, and Eq. 10 are higher than the upper bound limit of Pesaran et al. (2001) at 1% level of significance, respectively. It implies that long-run affiliation exists between patients and deaths due to COVID-19 and the VIX.

**TABLE 3:** ARDL bound test results (cointegration).

Equations	Model(lag)	F-statistic	I(0) at 1%	I(1) at 1%
Equation 6	ARDL(1, 3, 3, 3, 2, 1, 3, 1, 3)	19.3	2.62	3.77
Equation 7	ARDL(3, 3, 2, 0, 3, 3, 0, 0, 3)	8.7	2.62	3.77
Equation 8	ARDL(2, 3, 3, 3, 3, 3, 3, 3)	6.1	2.73	3.9
Equation 9	ARDL(4, 2, 1, 1)	8.61	3.65	4.66
Equation 10	ARDL(1, 1, 1, 1, 0, 1)	8.67	3.06	4.15
**Decision**				

*All models are cointegrated at 1% level of significance.Author’s calculation*

### The ARDL Model Considerations

The long- and short-run upshots of model A are demonstrated in Table 4. The short-run parameters showed that ITC had a positive impact on the VIX. At the same time, ITD showed a negative influence on the VIX, but the first and second lag of ITD rendered a positive influence on the VIX. These findings implied that the lockdown condition and health crisis in Italy, because of COVID-19, played a significant role in expanding the United States’ financial instability. The SPC and first lag value of SPC expressed a positive association with the United States markets’ financial volatility. Still, the second lag value of SPC had a negative relationship with financial stability. The health crisis in Spain (SPD) confirmed negative linkage with the uncertainty, although the first lag value of SPD directly affected the financial vulnerability in the United States. Shehzad et al. (2020a) elaborated that increasing COVID-19 cases and deaths have greatly damaged the market value of the S&P 500 and Nasdaq composite index.

**TABLE 4:** Long-run and short-run parameters of model A.

Variable	Coefficient	Standard error	*t*-Statistic	Probability	Variable	Coefficient	Standard error	*t*-Statistic	Probability
**Short-run**					D(GRC)	–0.437052	0.025498	–17.140	0
D(ITC)	0.108358	0.0062	17.314	0	D(GRD)	110.3016	6.229438	17.706	0
D[ITC(−1)]	0.084353	0.0046	18.138	0	D[GRD(−1)]	–342.3545	19.53159	–17.528	0
D[ITC(−2)]	0.096149	0.0060	15.872	0	D[GRD(−2)]	–333.8304	19.44324	–17.169	0
D(ITD)	–0.501345	0.0415	–12.062	0	D(UKC)	0.268873	0.051735	5.1971	0
D[ITD(−1)]	2.778611	0.1868	14.868	0	D(UKD)	47.12621	2.722459	17.310	0
D[ITD(−2)]	7.120248	0.4008	17.764	0	D[UKD(−1)]	30.7398	1.783848	17.232	0
D(SPC)	–0.160196	0.0116	–13.773	0	D[UKD(−2)]	24.69249	1.607525	15.360	0
D[SPC(−1)]	–2.20266	0.1252	–17.591	0	ECT_*t*_−_1_	–0.232007	0.013497	–17.189	0
D[SPC(−2)]	1.841172	0.1022	18.003	0	R-squared	0.986419			
D(SPD)	–0.980326	0.0797	–12.298	0	Adjusted R-squared	0.976495			
D[SPD(−1)]	46.90714	2.6982	17.384	0	Durbin-Watson	2.238767			
**Long**-**run**									
ITC	0.126448	0.0523	2.4173	0.02	GRD	1414.319	551.7185	2.5634	0.02
ITD	–19.5492	8.5399	–2.2891	0.03	UKC	–3.907005	1.392093	–2.8065	0.01
SPC	5.587578	2.4497	2.2809	0.03	UKD	632.6026	214.79	2.94521	0.00
SPD	–199.8744	78.205	–2.5557	0.02	C	19.53222	1.409447	13.8580	0
GRC	–3.192454	1.0700	–2.9834	0.00					

*Author’s calculation.*

Moreover, GRC reported a negative association with the VIX, and GERD revealed a positive impact on the VIX. Further, UKC and UKD had an encouraging impact on the VIX. ITC, SPC, GERD, and UKD made a positive and significant impression on the VIX in the long-run. It signifies that the economic crisis and health crisis in Italy and Spain negatively effected the United States’ financial uncertainty for the long-run period. Furthermore, the health crisis in Germany and the United Kingdom substantially destabilized the financial stability of the United States. However, ITD, SPD, GRC, and UKC pointed out a distinct effect on financial volatility in the United States. These statistics imply that an intensification in lockdown circumstances and health crisis owing to COVID-19 occupy a substantial part in escalating the United States’ financial instability.

Besides, the negative and significant coefficient of ECT_*t–1*_ indicated that aberration in the financial stability today will move back to equilibrium with the adjustment speed of 23% tomorrow. Alola and Olowu (2020) analyzed the impact of COVID-19 on pharmaceutical product inflation. The study revealed that uncertainty arising from the pandemic was more remarkable than economic uncertainty in the United States. The study also documented that total spillover had been increased from 34.2 to 47.6% due to COVID-19 uncertainty. Alola and Bekun (2020) stated that disposable income per capita (DIC), crude oil (WTI), the electric power sector (EEM), and industrial sector (IEM) faced a net spillover shock of 25.90, 63.80, 26.10, and 48.80%, respectively, out of the total spillover index.

The regression parameters of model B are available in Table 5. These parameters illustrated that an expanding number of patients of USC performed a crucial role in building the financial volatility of their markets. However, the death rate in the United States (USD) and the Netherlands (NETD) showed a reverse impact on financial volatility. Moreover, the abundance of confirmed cases in France (FRC) positively and severely impacted the fear gage. The results of long-run coefficients designated that lockdown condition and health crisis in Switzerland (SWC and SWD) expressively upsurged the uncertainty level of the United States financial markets.

**TABLE 5:** Long-run and short-run parameters of model B.

Variable	Coefficient	Standard error	*t*-Statistic	Probability	Variable	Coefficient	Standard error	*t*-Statistic	Probability
**Short-run**									
D[VIX(−1)]	0.320184	0.071133	4.5012	0.00	D(NETD)	–37.1194	3.477211	–10.67	0
D[VIX(−2)]	0.433447	0.091629	4.7304	0.00	D[NETD(−1)]	–154.4663	13.54778	–11.40	0
D(USD)	–6.027666	0.455287	–13.239	0	D[NETD(−2)]	–128.413	10.72135	–11.97	0
D[USD(−1)]	–15.10811	1.35129	–11.180	0	D(FRC)	0.581531	0.049238	11.810	0
D[USD(−2)]	–10.79734	1.108114	–9.7438	0	D[FRC(−1)]	1.182217	0.104662	11.295	0
D(USC)	0.152815	0.013592	11.2426	0	D[FRC(−2)]	0.785776	0.069238	11.348	0
D[USC(−1)]	0.192244	0.015738	12.2155	0	ECTt-1	–0.337813	0.029974	–11.27	0
D(SWC)	–0.633202	0.056824	–11.143	0	R-squared				
D[SWC(−1)]	–3.696133	0.32525	–11.363	0	Adjusted R-squared				
D[SWC(−2)]	–2.134	0.189695	–11.249	0	Durbin-Watson				
**Long-run**					NETD	19.85601	61.55645	0.3225	0.75
USD	40.38965	17.49182	2.3090	0.0317	NETC	–12.63964	6.054786	–2.087	0.05
USC	–0.439103	0.143477	–3.060	0.0062	FRD	39.32385	17.16386	2.291	0.03
SWD	63.43783	31.56593	2.0096	0.0581	FRC	–0.952051	0.427182	–2.228	0.03
SWC	11.93463	4.233947	2.8187	0.0106	C	17.22974	1.53559	11.220	0

*Author’s calculation.*

Further, USD, NETD, and FRAD also raised the financial uncertainty of the United States markets considerably. However, USC, NETC, and FRC displayed negative bonding with the VIX, which means lockdown and economic crisis in the United States, the Netherlands, and France had a domineering role in changing the financial stability in the United States. The negative and significant value of ECT_*t–1*_ reported that the disequilibrium of the VIX would be rectified with a speed of 33% each day. Alola et al. (2020) analyzed the impact of COVID-19 deaths and recovery on financial stress in the United States. The study used the Markov switching method and found that recovery from COVID-19 significantly reduced financial stress, while an increase in deaths due to COVID-19 fettered the United States’ financial stress.

The verdicts of the Asian nations are shown in Table 6. These results showed that the first and second lag values of confirmed China cases (CHC) had an absolute sway on the financial instability index. Moreover, the first and second lag of CHD publicized a negative impression on the VIX. The lockdown due to COVID-19 in Japan (JAPC) ominously raised the financial markets’ impulsiveness, but JAPD pointedly supported the United States’ financial stability. Nonetheless, confirmed COVID-19 cases and deaths in Iran (IRC and IRD) conveyed a significant and positive nexus with variance in the United States’ financial markets. Besides, confirmed cases of COVID-19 in Pakistan (PAKC) also meaningfully amplified the uncertainty of financial markets in the United States. The long-run coefficients of Asian nations intimated that CHC limited VIX, but CHD and JAPD significantly enlarged the financial system’s shakiness in the United States.

**TABLE 6:** Long-run and short-run parameters of ARDL model C.

Variable	Coefficient	Standard error	*t*-Statistic	Probability	Variable	Coefficient	Standard error	*t*-Statistic	Probability
**Short-run**									
D[VIX(−1)]	–0.121317	0.07492	–1.61	0.15	D(IRC)	0.025592	0.00486	5.265	0.00
D(CHNC)	–0.000835	0.00029	–2.84	0.02	D[IRC(−1)]	0.047561	0.007196	–6.609	0.00
D[CHNC(−1)]	0.002225	0.00036	6.085	0.00	D[IRC(−2)]	0.041762	0.00475	–8.791	0.00
D[CHNC(−2)]	0.002097	0.00039	5.338	0.00	D(IRD)	0.220182	0.055997	3.932	0.00
D(CHND)	0.025387	0.02166	1.171	0.28	D[IRD(−1)]	0.323953	0.080449	4.026	0.00
D[CHND(−1)]	–0.049799	0.02089	–2.38	0.05	D[IRD(−2)]	0.353056	0.118423	–2.981	0.02
D[CHND(−2)]	–0.074943	0.01340	–5.59	0.00	D(PAKC)	0.123498	0.024374	5.066	0.00
D(JAPC)	0.126562	0.03972	3.185	0.01	D[PAKC(−1)]	0.086263	0.03167	2.723	0.03
D[JAPC(−1)]	0.215088	0.04080	5.271	0.00	D[PAKC(−2)]	0.104221	0.028715	3.629	0.01
D[JAPC(−2)]	0.141362	0.04823	2.93	0.02	ECTt-1	–0.718286	0.0633	–11.34	0.00
D(JAPD)	1.042896	0.53948	1.933	0.10	R-squared	0.970102			
D[JAPD(−1)]	–3.581338	0.80170	–4.46	0.00	Adjusted R-squared	0.923118			
D[JAPD(−2)]	–4.013693	0.78239	–5.13	0.00	Durbin-Watson	2.052251			
**Long-run**									
CHNC	–0.00502	0.00062	–8.00	0.00	IRC	0.048397	0.008973	5.393	0.00
CHND	0.078293	0.03507	2.232	0.06	IRD	–0.416392	0.171506	0.427	0.51
JAPC	–0.292012	0.17807	–1.63	0.15	PAKC	0.051902	0.125891	0.412	0.69
JAPD	15.83017	3.28179	4.823	0.00	C	26.98563	2.103817	12.82	0.00

*Author’s calculation.*

Also, IRC ameliorated VIX; however, IRD had an insignificant impact on VIX. These statistics implied that the amplification of the lockdown situation in Asian nations due to COVID-19 had a more significant impact on the United States financial market’s volatility. The ECT_*t–1*_ coefficient pronounced that disequilibrium ensued in the VIX today, due to COVID-19 in Asian nations, would move back to equilibrium tomorrow, with an adjustment speed of 71%, meaning that Asian crises due to COVID-19 would rapidly off-set the United States financial market’s volatility. Balsalobre-lorente (2020) argued that in spite of the isolation of China from the world, there was an increase in its economic growth.

Table 7 manifests the results of COVID-19 in Arabian nations. The findings discovered that the economic crisis in Qatar (QTRC) owned an immediate position in mounting the volatility of financial markets, for both long-run and short-run periods. Also, UAEC had a positive and significant dominance on the VIX. On the other hand, lockdown in Saudi Arabia (SAC) enhanced instability in the long-run. The significant value of ECT_*t–1*_ signified that the unbalance of the VIX, due to COVID-19’s impact in Arabian nations, would become stable with an adjustment speed of 42.8%. The consequences related to the COVID-19 effect in African nations are given in Table 8. These factors showed that the economic crisis in Ghana (GHNC) and Kenya (KENC), due to COVID-19, significantly affected the United States financial markets’ volatility.

**TABLE 7:** Long-run and short-run parameters of ARDL model D.

Variable	Coefficient	Standard error	*t*-Statistic	Probability	Variable	Coefficient	Standard error	*t*-Statistic	Probability
**Short-run**					D(SAC)	–0.129345	0.019802	–6.53185	0.00
D[CLOSE(−1)]	–0.541458	0.11249	–4.8	0.00	D(UAEC)	0.170104	0.030372	5.600676	0.00
D[CLOSE(−2)]	–0.806096	0.14811	–5.4	0.00	ECTt-1	–0.428924	0.046235	–9.27712	0.00
D[CLOSE(−3)]	–0.328253	0.09702	–3.3	0.00	R-squared	0.971788			
D(QTRC)	0.064296	0.01081	5.94	0.00	Adjusted R-squared	0.947102			
D[QTRC(−1)]	0.1299	0.01793	7.2	0.00	Durbin-Watson	2.2658567			
**Long-run**									
QTRC	0.422218	0.11500	3.67	0.02	UAEC	1.091373	0.461971	2.362427	0.07
SAC	–0.503365	0.21419	–2.3	0.07	C	65.61467	5.787498	11.33731	0.00

*Author’s calculation.*

**TABLE 8:** Long-run and short-run parameters of ARDL model E.

Variable	Coefficient	Standard error	t-Statistic	Probability	Variable	Coefficient
**Short-run**						
D(EGPC)	0.164266	0.099597	1.649304	0.1976	R-squared	0.893278
D(GHNC)	1.943076	0.253809	7.65567	0.0046	Adjusted R-squared	0.845846
D(KENC)	6.838584	0.964837	7.087815	0.0058	Durbin-Watson	2.691855
D(TUNC)	0.059362	0.134444	0.441533	0.6887		
ECTt-1	–0.729633	0.090354	–8.075309	0.004		
**Long-run**						
EGPC	–0.218354	0.285338	–0.765248	0.4998		
GHNC	–8.523562	2.784284	–3.061312	0.0549		
KENC	21.09007	6.97338	3.024368	0.0566		
MORC	4.011221	1.323988	3.029651	0.0563		
TUNC	2.428482	1.048609	2.315907	0.1035		
C	73.65115	4.75252	15.49728	0.0006		

*Author’s calculation.*

Nevertheless, GHNC showed a cynical alliance with VIX in the long-run. Besides, MORC and TUNC had a positive correlation with the VIX. The negative value of ECT_*t–1*_ suggested that disequilibrium in the VIX today, because of COVID-19 in African nations, would be balanced tomorrow with an adjustment speed of 72.9%. Shehzad et al. (2020b) analyzed and compared the impact of COVID-19 and the global financial crisis (2007–2009) for Europe, the United States, and Asia. The investigation revealed that the COVID-19 crisis has had more of an impact on these economies than the global financial crisis. Additionally, the investigation documented that the trade war between China and the United States remained insignificant in returns spillover.

### Diagnostic Measures

**FIGURE 3:**
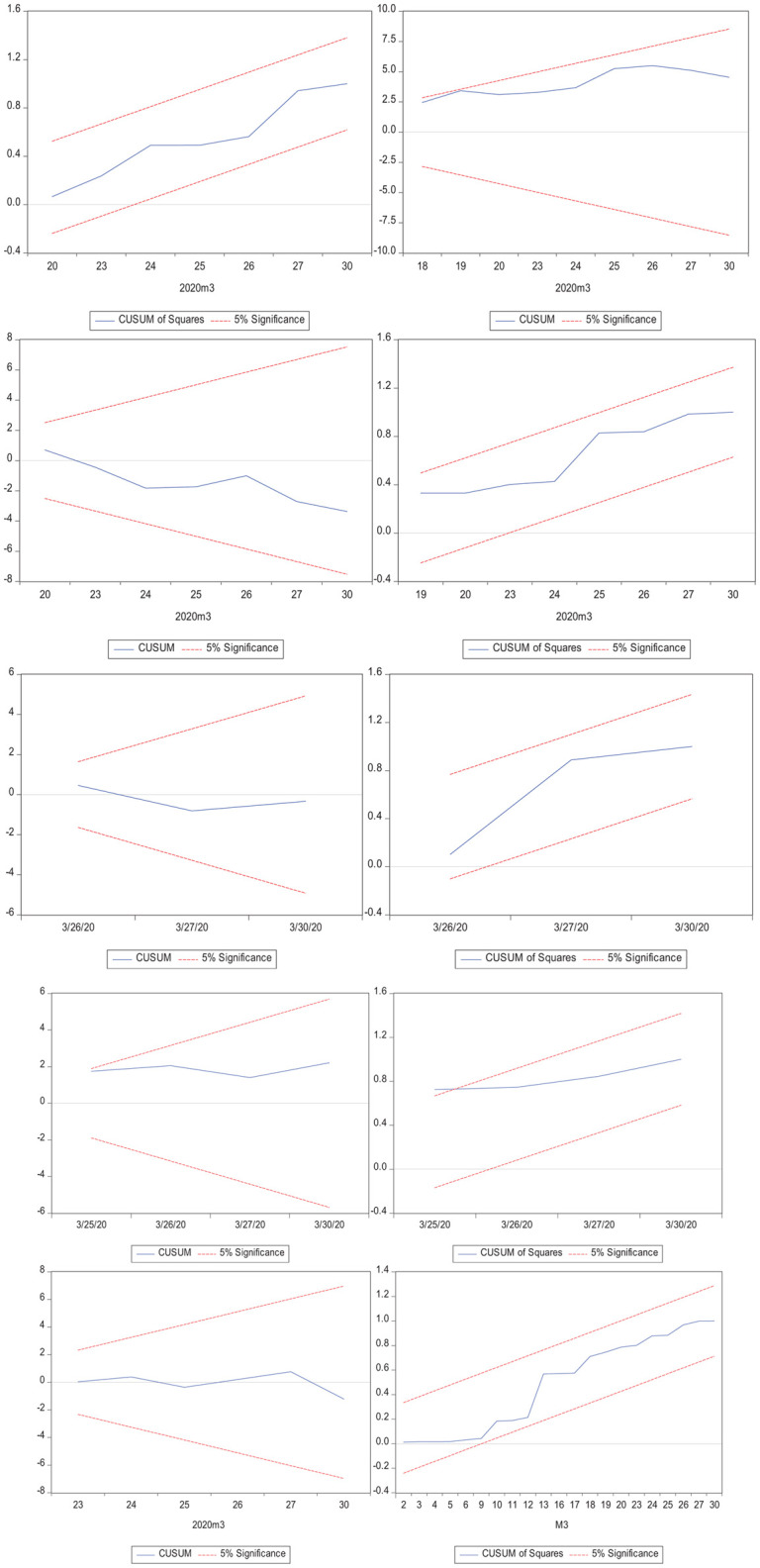
CUSUM and CUSUMSQ plots.

This investigation employed the Breusch–Godfrey (Breusch and Pagan, 2006a, b) model to assess the serial correlation and heteroskedasticity in the residuals in each model. Moreover, to verify the accuracy of the coefficients of each model, the CUSUM and CUSUMSQ tests (Brown et al., 1975) were used. Further, functional misspecification in each model was determined through the Ramsey analysis. The results of these diagnostic measures, shown in Table 9, clarify no serial correlation, heteroskedasticity, or functional misspecification in the models. Also, the parameters of each model were correct as the plots of CUSUM and CUSUMSQ, presented in Figure 3, were within the 5% critical limit.

**TABLE 9:** Findings of diagnostic techniques.

	Heteroskedasticity		Serial correlation		Ramsey test	
Model A	F-statistic	0.308853	F-statistic	0.454049	F-statistic	0.37281
Model B	F-statistic	0.091074	F-statistic	0.023862	F-statistic	0.025706
Model C	F-statistic	0.362872	F-statistic	1.266271	F-statistic	2.254878
Model D	F-statistic	1.097822	F-statistic	0.241042	F-statistic	0.56118
Model E	F-statistic	0.642662	F-statistic	0.433606	F-statistic	0.257286

*Author’s calculation.*

## Conclusion

The novel coronavirus pandemic (COVID-19) has significantly affected the economy of the world. Moreover, the world’s stock markets faced collapse in their market values, and crude oil prices in the United States become negative due to lockdown. The principal aim of this project was to determine the impact of the economic and health crisis generated by USC on its financial stability. This study also evaluated the spillover effects of the economic and health crisis, transpired because of COVID-19 in the European, African, Asian, and Arabian nations, on the United States’ financial stability. For the said purpose, this investigation utilized the ARDL model. The investigation observed that economic crisis occurring because of COVID-19 in Italy and Spain expressively affects the United States’ financial instability for the short-run period. However, in the long-run, it improves the financial stability of the United States markets.

Furthermore, lockdown in the United Kingdom and Germany negatively affects the financial stability of the United States markets. The analysis signified that the health crisis in the United States due to COVID-19 meaningfully escalated the instability of the United States financial markets for the long-run period. Also, the health crisis in France and the Netherlands due to COVID-19 showed a harmful impact on the United States’ financial stability. Conversely, the economic crisis in France and the Netherlands limited the instability of the United States markets. The model of Asian nations implied that the economic crisis of China, Japan, Iran, and Pakistan significantly and positively contributed to the United States’ financial instability.

Nonetheless, lockdown in Qatar and UAE indicated a destructive influence on the financial stability of the United States. Still, lockdown in Saudi Arabia denoted an insignificant impact on the United States financial system instability. Additionally, in the short-run, the economic crisis of Ghana, Kenya, Morocco, and Tunisia tremendously raised the financial instability of the United States markets, but, in the long-run, Ghana’s economic crisis improved the financial stability of the United States. This study argued that disequilibrium occurred in the United States’ financial VIX due to COVID-19’s impact in Europe, and the United States adjusted deliberately as opposed to other evaluated nations. Moreover, the deviation transpired in the United States’ financial stability, due to COVID-19 effects in Asian and African nations, achieved its equilibrium rapidly. The investigation findings confirmed that the economic crisis that occurred in the world significantly distressed the financial stability of the United States markets. The health crisis that occurred in the United States affected the United States markets’ financial stability compared to the health crisis that occurred in other nations of the world. Hence, additional resources must be allocated to the health sector to mitigate such epidemic or pandemic situations in the future. The study inferred that lockdown due to COVID-19 had damaged the supply and demand cycle; thus, economic conditions had become uncertain all around the globe. Hence, it had dramatically affected all kinds of business activities, specifically, small businesses. The government should build novel policies to support small businesses, such as reducing taxes and other duties. The investigation suggests that the United States and other nations should build strategies of smart lockdown to begin the economic cycle. Likewise, online business and home delivery services should be permitted. Additionally, the use of digital currency can play an imperative role in smart lockdown. Further, there should be a cut on the central banks’ interest rates and such policies should mainly focus on small investors.

Finally, this article aimed to contribute to the literature by examining the role of the economic crisis and health emergencies caused by USC, Europe, Arabian, African, and Asian countries in the United States’ financial crisis. However, the article has some limitations. We focused on the impact of the COVID-19 pandemic on the financial system. Future research may examine the impact of the pandemic on the real economy. The nexus between health shock and supply-demand mechanisms can be discussed. For instance, the dynamic effects of the COVID-19 outbreak on tourism, energy, and food demands can be explained. To fight COVID-19, this type of research focusing on the socio-economic effects of the pandemic and vaccination and treatment options is desperately needed.

## Data Availability Statement

The original contributions presented in the study are included in the article/supplementary material, further inquiries can be directed to the corresponding author/s.

## Author Contributions

LX: conceptualization, data curation, formal analysis, investigation, writing – original draft, and supervision. FB: conceptualization, data curation, writing – original draft, and writing – review and editing. EK: data curation, methodology, formal analysis, and writing – original draft. All authors contributed to the article and approved the submitted version.

## Conflict of Interest

The authors declare that the research was conducted in the absence of any commercial or financial relationships that could be construed as a potential conflict of interest.

## Footnotes

1 This study excluded the incomplete data of the confirmed number of deaths due to COVID-19 in Pakistan, Saudi Arabia, UAE, Qatar, Egypt, Morocco, Kenya, Tunisia, and Ghana.
